# Application of the Logic Model to the School-Based Fit and Smart Adolescent Smoking Cessation Programme

**DOI:** 10.21315/mjms2022.29.5.14

**Published:** 2022-10-28

**Authors:** Nur Atikah Abdul Halim, Lei Hum Wee, Nur Zakiah Mohd Saat, Swinderjit Jag Jit Singh, Ching Sin Siau, Caryn Mei Hsien Chan

**Affiliations:** 1 Centre for Community Health Studies (ReaCH), Faculty of Health Sciences, Universiti Kebangsaan Malaysia, Kuala Lumpur, Malaysia; 2 National Cancer Society Malaysia (NCSM), Kuala Lumpur, Malaysia; 3 School of Medicine, Faculty of Medical and Health Sciences, Taylor’s University, Selangor, Malaysia

**Keywords:** smoking cessation programme, intervention, adolescent, logic model, school-based

## Abstract

**Background:**

School-based smoking cessation intervention programmes are challenging to implement and evaluate. This study aimed to explain the process of developing the Fit and Smart Adolescent Smoking Cessation Programme (FSSCP). Logic model is a visual tool that helps programme planners to create an activity action plan that suits the target group to achieve programme objectives and goals.

**Methods:**

This two-arm cluster-randomised controlled trial was implemented between January 2018 and November 2018. Six schools were selected using stratified random sampling, whereby students were purposively selected and invited. The criteria of inclusion to the programme were secondary school students (aged 13 years old–17 years old) who smoked conventional cigarettes (CC) and electronic cigarettes (EC).

**Results:**

A total of 422 students from six schools participated in this study. Three schools were designated as intervention (*n* = 250) and the other three as control schools (*n* = 172). Formative evaluation of participants in the FSSCP using the logic model showed that participants were satisfied with the overall programme (91.5%), were motivated to stop smoking (90.4%) and were prevented from relapse (89.2%). The quit rate at a 3-month follow-up was 41.8%.

**Conclusion:**

The logic model supported the development of the programme, with details on the processes, dissemination activities, identification of barriers, evaluation criteria and outcomes provided.

## Introduction

There is an increasing trend in usage of conventional and e-cigarettes among adolescents in Malaysia. Globally, 9.4% (1) rate of smoking among adolescents in the 13-year-old to 15-year-old age bracket has been reported. In Malaysia, the rate is much higher at 14.8% (2). Adolescent smoking can lead to tobacco addiction and early smoking uptake that is related to the risk of dependence in adulthood (3). Currently there are three smoking prevention initiatives in Malaysia: IMFree (4), Say No to Smoking campaign (5, 6) and Young Doctor (4) but there is currently no empirically established school-based smoking cessation intervention programme (7).

An effective adolescent smoking cessation programme should be carefully designed in the early stages that is tailored for target population and emphasises on factors that promote smoking initiation and maintenance (8). A review on tobacco cessation interventions among adolescents revealed that school-based programmes are often challenging to implement and hard to interpret due to limited descriptions of the programme’s planning and evaluation (9). In addition, most of the studies reviewed suffered from high or unclear risk of bias, clinical heterogeneity and imprecise methods (9). Adolescents reported that quitting tobacco is not difficult enough to warrant professional assistance. Furthermore, they downplayed the addictiveness of tobacco and did not have adequate interest to participate in the cessation interventions (8, 10, 11), further complicating the validation of these programmes.

Developing a school-based smoking cessation intervention requires careful planning (12, 13), clear goals and continued reported satisfaction of participants and funders (12, 14–16). To be more effective, the interventions need to take into account multiple factors so that it is multi-component, comprehensive and synergistic with a supportive environment (8, 17–19).

The logic model may provide a productive framework for effective planning, suggested by the theory of change and ability to evaluate accurately its processes and outcomes (12, 20, 21). A logic model is a graphic illustration of how a programme or intervention is expected to produce desired outcomes (13, 22–24). It shows the relationships among the inputs, available resources, how to deliver an intervention from a series of activities and expected results (13, 25, 26). This model is also known as a programme model (13) and the blueprint for the programme (25, 26). The use of a logic model is advantageous over other intervention planning methods (that also include community and stakeholder engagement) as it is a more comprehensive and systematic intervention planning method (22, 24, 26, 27).

The paper aims to illustrate the process of developing the logic model of a school-based smoking cessation programme, the FSSCP, from the planning to the implementation and evaluation stages.

## Methods

### The Fit and Smart Adolescent Smoking Cessation Programme

The Fit and Smart Adolescent Smoking Cessation Programme (FSSCP) is a school-based multi-component approach to assist adolescents (aged 13 years old–17 years old), who smoke conventional cigarette (CC) and electronic cigarette (EC), to quit smoking. The FSSCP was established in the Faculty of Health Sciences, Universiti Kebangsaan Malaysia (UKM) in collaboration with the National Cancer Society of Malaysia (NCSM) as well as three selected schools (intervention schools) in 2018.

The relationship between UKM, NCSM and other schools was developed through a series of discussions to explain the scope of research and cooperation needed before they agreed to participate directly. Partnership with the NCSM was important as this organisation is the first non-profit cancer organisation in Malaysia and is actively providing education, care and support services for individuals affected by cancer and general public. Partner schools provide manpower (counsellors) and infrastructure support for service delivery such as intervention rooms and internet access.

Designing a school-based smoking cessation intervention programme using a logic model could aid in the planning process of the programme and inform its implementation (28, 29). Therefore, we extended the involvement of stakeholders by presenting FSSCP at the ministry level in the Malaysian government. The Tobacco Control Sector of the Ministry of Health (MOH) Malaysia and the Ministry of Education (MOE) were involved by providing consultation on the framework of intervention to ensure that the training module is aligned with the existing mission, vision and priority areas of the adolescent stop smoking strategies of the respective ministries. The endorsement of the MOH will allow FSSCP module to be employed to train healthcare providers nationwide. The involvement of participating schools is equally important to ensure all activities planned for the students are aligned with the schools’ learning and teaching principles.

In embarking on this programme, the non-governmental stakeholder (NCSM) was also involved and trained to provide them the capacity to continue implementing the programme in other schools after assisting in this current programme.

#### The Process of Logic Model Development for Fit and Smart Adolescent Smoking Cessation Programme

**Figure 1:**
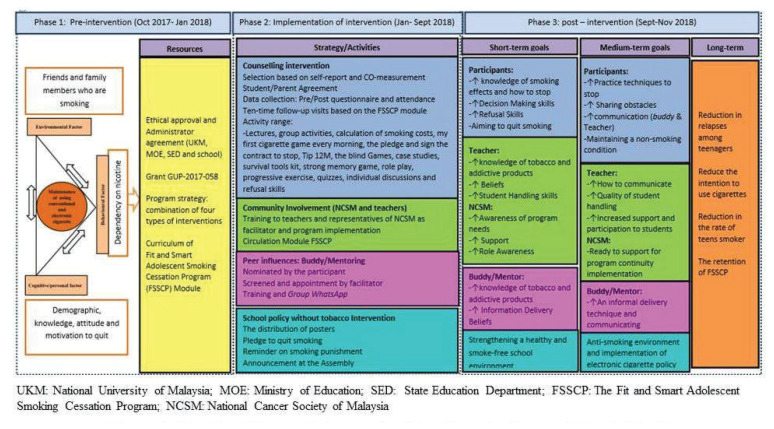
The FSSCP’s logic model

Overall, the process of developing and finalising the logic model of FSSCP was implemented in three phases: i) planning, ii) implementation and iii) evaluation (Figure 1). Development of a working relationship with the stakeholders such as the NCSM, MOE Malaysia, the school administrative authorities and teachers was important. During the planning, implementation and evaluation stages, an attitude of inclusion and respect was dominant to build the trust and relationship with the stakeholders.

From the pre-intervention preparations, the research team held a series of discussions with the NCSM. The discussion led to the development of research proposal papers and ethics applications with stakeholders such as the MOE Malaysia and school administrative authorities. Receiving support from the school principals and teachers in the pre-intervention stage was of the utmost importance to ensure the success of the programme (30–32).

The selection of participating schools was decided in this phase. This was a two-arm cluster-randomised controlled trial, implemented between January 2018 and November 2018. Two levels of sampling were employed: schools were selected using stratified random sampling, whereas the students were purposively selected and invited, and student participants were purposively recruited, who had engaged in tobacco products. The objective of this study was to evaluate the effectiveness of programme where quit rate as well as formative assessments were evaluated. A total of 6 out of 89 secondary schools were selected randomly from all zones in the Federal Territory of Kuala Lumpur, with the ratio of two schools for each of the three zones (Bangsar/Pudu, Sentul and Keramat). In order to detect a small effect size of Cohen’s *d* = 0.40 with 95% power (alpha = 0.05, two-tailed), G*Power suggests a minimum of 328 participants (164 per arm) in an independent samples *t*-test (33). Taking into account a 10% dropout rate, a total of 180 participants per arm were required.[Fig f2-14mjms2905_oa]

### Phase 1: Problem identification and planning

The main objective of this phase was to define the primary health issue and its determinants. This phase involved brainstorming effective strategies for adolescent smoking cessation development of FSSCP’s training modules and survey measure. A work plan (Table 1) and logic model draft for FSSCP were developed by the research team and presented during the primary meeting.

The primary meeting was held to explain the objectives, three major components of logic model for FSSCP (inputs, outputs and outcomes), programme planning and training using the FSSCP module. This meeting was attended by four representatives from UKM, five representatives from NCSM and counsellors from four schools. The presence of these representatives was important as they were the implementers who would provide valuable input due to their greater awareness of the school atmosphere (34–36).

An agreement was reached at this meeting, which included not using sensitive words or insist that there was a misuse of tobacco among students. Since a programme or study at a school would be deemed more acceptable if it avoided sensitive issues (37), the original programme name was thus re-branded as FSSCP, so as to display a more positive image and avoid any stigma that may arise among parents and school community (teachers and students).

Student participants aged 13 years old–17 years old were purposively recruited. These students included those who had either smoked CC, EC and/or shisha for the last 30 days; this was either self-declared or identified by the students’ discipline teachers. Written consent was obtained from the participants, their parents and schools, and then pre-data collection was conducted. The smoking status of adolescents was determined based on the question, “Have you ever used: i) conventional cigarettes only, ii) electronic cigarettes only and iii) a combination of conventional and electronic cigarettes?” The adolescents who answered that they only used conventional cigarettes were categorised as the sole CC users, those who only used electronic cigarettes were the sole EC users and those who used both CC and EC were the dual users (38). The hooked-on nicotine checklist (HONC) (39, 40) questionnaire was used to determine the onset and strength of tobacco dependence of CC and EC by adding EC element to the original version. A HONC score of ≥ 1 indicated that a participant was hooked on smoking. The Malay translated version of the HONC reported an internal consistency reliability Cronbach’s α of 0.924.

### Phase 2: Programme implementation

The second phase was the implementation phase, which consisted of 10 school visits. The strategies of the FSSCP, a multi-component intervention, were developed based on extent literature (8, 15, 16). As stated in the planning stage, the implementation strategy of these activities had been agreed upon by all parties. The implementation of the programme consisted of four intervention components: i) counselling, ii) peer influence, iii) community involvement and iv) implementation of tobacco-free school policy. This combined approach has been shown to yield great success in reducing or altogether preventing nicotine addiction among high school students (15, 41, 42).

The counselling intervention was included in accordance with the principles of social cognitive theory and was adapted based on the Quit4Life (Q4L) programme (15, 43). The counselling component was managed by school counsellors who were trained and had experience in handling smoking cessation (17). Every session lasted between 45 min and 60 min, and covered topics regarding motivation and preparation to quit, getting social support and maintaining smoke-free conditions. The peer intervention approach was implemented by allowing students who smoked to choose a non-smoking peer, known as a ‘buddy’, in the 5th session. Peers can have a significant impact in helping those who smoke overcome the challenges faced while in the process of quitting smoking (15, 44). In addition, peer influence plays a very important role because at this stage, school students are more likely to spend time with peers. All selected buddies underwent training to assist their smoking peers by utilising buddy help-smoking cessation diaries. Community involvement was used to gain social support from teachers and non-governmental organisations. In this programme, support from the participating schoolteachers and the NCSM was received, where they acted as facilitators. The involvement of these stakeholders strengthened social support and policy-making, thus providing a positive systemic environment for efforts in quitting smoking (17). The last intervention domain was strengthening the tobacco-free school policy for all students, teachers, staff and visitors through talks during school assemblies and display of banners and posters. Ownership or use of tobacco products by students, teachers and staff was prohibited at all times on school grounds, in vehicles or at any school events (whether on or off-premises). Tobacco-free school policy would thus be effective in helping the students in maintaining a smoke-free environment (45).

In this implementation phase, the research team identified the crucial issues that were important in revising the original FSSCP’s logic model. Several challenges rose throughout the implementation process that involved ten visits to each school, negative perceptions of subject teachers towards FSSCP sessions, barriers which might have prevented them from attending these sessions and attitude of participants who merely attended programme sessions so that they could skip classes. Furthermore, attendance rates per session seemed to decrease with each subsequent session. Finally, the lack of sufficient staff to conduct these sessions in addition to the large numbers of participants also posed challenges.

### Phase 3: Post-intervention

**Figure 3:**
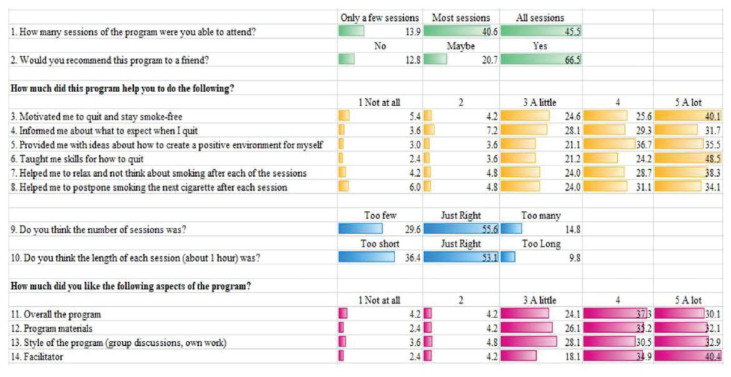
Formative evaluation for the FSSCP Note: Participants’s feedback (*N* = 168)

This phase was the post-intervention phase whereby the FSSCP was evaluated by teachers (in-depth interviews with counsellors, senior assistant of student affairs and disciplinary teachers) and the students who participated in this programme. Exhaled CO level was measured using MicroCO meter to objectively identify the participants’ smoking status. Those with CO levels of 4 ppm–6 ppm were categorised as light smokers and those with levels ≥ 7 ppm as regular smokers. However, only descriptive analyses and a formative evaluation (4 weeks) by students were reported (Figure 3).

Descriptive analysis was conducted using IBM SPSS Statistics for Windows, version 25.0 (SPSS Inc., Armonk, NY), with pre- and post-intervention numbers and percentages broken down for study participants by sociodemographic characteristics (for example, age, gender and monthly household income). Participants’ evaluation of the programme was tabulated. In-depth interviews were also conducted with the senior assistant of student affairs, counsellors and disciplinary teachers from the schools who participated in the intervention but has not been reported in this paper.

The FSSCP’s impact evaluation and final report was prepared and presented to FCTC unit and MOH for retention and sustainability of FSSCP. Several presentations were also carried out to the stakeholders, which were the task force committee (MOH), the daily school management division (MOE), the Minister of Health (MOH), Chairperson for electronic cigarettes and vaping task force (MOH) and the Deputy Minister of Education (MOE).

## Results

A total of 422 students from six schools participated in this study. Three schools were designated as intervention (*n* = 250) and the other three as control schools (*n* = 172). At pre-intervention, most of the participants were 16 years old (29.1%), male (90.3%), had monthly household income of RM1,001–RM4,000 (82.2%) and initiated smoking between 13 years old and 17 years old (59.0%). Most of them were sole-EC users (42.2%) (Table 2).

The quit rate at the post-intervention phase was 56.5% at the 4th week (CO-validated). The quit rate was 41.8% (CO-validated) and 52.9% (salivary cotinine-validated) at the 3-month follow-up. After being tested for CO expired air, the participants were immediately asked to provide a saliva sample to be tested with the nal von minden Drug-Screen® Saliva Classic Test (Tables 2 and 3).

Formative evaluation indicated that 84.5% of the participants attended all ten sessions of the programme. A total of 64.9% participants responded that the overall programme was ‘liked a lot’ and that they were willing to recommend the programme to their friends. Almost 90% of the participants indicated that they liked the following aspects of the programme: the overall programme, programme materials, approach (group discussions, role play and personal sharing) and facilitators.

## Discussion

This study used the logic model as a framework and enabled the refinement of each process in the planning, development, implementation, monitoring and evaluation of FSCCP (10, 13, 18–20). This study also revealed contradictory findings with the current literature which indicate that adolescents are reluctant to participate in group interventions (21–23), especially interventions that are perceived as sensitive, such as substance use. As revealed in the formative evaluation results, most participants attended the whole programme and reported high liking ratings of the programme, which may indicate that the programme met their needs (24).

Almost all participants indicated that they liked important aspects of the programme such as its materials, approach and facilitators. This was in line with previous studies (19, 20, 25, 26) which stated that an effective programme implementation was influenced by the characteristics of the organisation providing the programme and its facilitators (20). More than half of the participants (53.1%) agreed that the number of sessions and duration of each session (about 1 h) (55.6%) was just right. Therefore, in future implementations of the programme, this will be continued to ensure that the programme suits the needs of participants, whereby the programme is not too complex or too lengthy, in order to obtain the best results (19).

With high commitment from all parties involved (participants, facilitators and school community), this programme was able to achieve its short-term and intermediate-term goals. The participants reported that the programme increased their motivation to quit, provided them with information about quitting, created a positive environment for quitting and improved their coping skills. The participants’ responsiveness may have influenced the outcomes and quality of programme implementation (19). This would indeed be important for the long-term goal of sustaining FSSCP.

Implementing programmes in schools that involve adolescents is never without challenges. Among the three schools that received intervention, two schools conducted the programme during school hours and one school conducted it after school hours. A few teachers did not allow their students to attend the programme during their lessons. We noticed that this happened due to the lack of programme’s publicity and the lack of awareness among the teachers concerning the programme, which caused them to be reluctant to cooperate. As a result, we have improved the original logic model with the addition of the following elements: i) every school community be informed that the programme is to be implemented within a specific time frame, ii) a focus group (without disclosing sensitive matters) be selected and iii) incentives be provided throughout the session. Therefore, the graduation day (at the very end of the programme) would be an important occasion for celebrating students who have successfully quit their use of electronic cigarettes and conventional cigarettes.

However, we were faced with lower attendance rates particularly when the programme was conducted after school hours, whereby most students would go home immediately after their final class for the day. In addition, there was a lack of facilitators to conduct a number of group activities during sessions, despite our prior cooperation with NCSM. Therefore, the most important key point for the implementer is to ensure that the involvement of school committee is at the very start of the programme to develop a sense of belonging, rapport and commitment between the parties involved (24).

### Implications for School-Based Smoking Cessation Programmes

Recommendations for researchers who wish to use a logic model framework for school-based smoking programme evaluation include:

Place greater emphasis on the perspectives of teachers, school administrators, parents, participating students and stakeholders throughout the entire development and implementation of the programme.

Integrate evaluations into the programme planning from the very beginning.

Give consideration to the implications of manpower and funding. This would also help in addressing issues of programme sustainability (24).

Keep abreast with best practice models in teenage smoking cessation programmes, especially regarding the new challenges faced in consumption and usage of tobacco products such as electronic cigarettes, heated tobacco products (HTP) (27).

Work with diverse communities to develop students’ trust and maintain confidentiality (24, 28).

### Limitations and Strengths

To the best of authors’ knowledge, the current study is the first in the area that utilises logic model in the process of planning, implementation and evaluation of a school-based smoking cessation programme targeted towards students who smoke. However, this study had a few limitations. Firstly, this study was conducted in the Federal Territory of Kuala Lumpur and therefore may not be generalisable to other states in Malaysia. Secondly, the authors were not able to validate the use of EC due to financial constraints and therefore depended on self-reports of the participants, unlike the use of CC, which was validated using CO readings. This may have led to inconsistencies in the smoking status reported. Lastly, the research team only conducted the 3-month follow-up at the schools that received intervention due to lack of manpower, money and time.

## Conclusion

The logic model may serve as a practical framework for stakeholders, which include school communities and healthcare providers, to plan, implement and evaluate school-based smoking cessation intervention programmes. This study also demonstrated that community input at the beginning of a programme ensures its relevance to community needs and gains stronger support, which would result in an implementation that is more likely to be attainable and sustainable.

## Figures and Tables

**Figure 2 f2-14mjms2905_oa:**
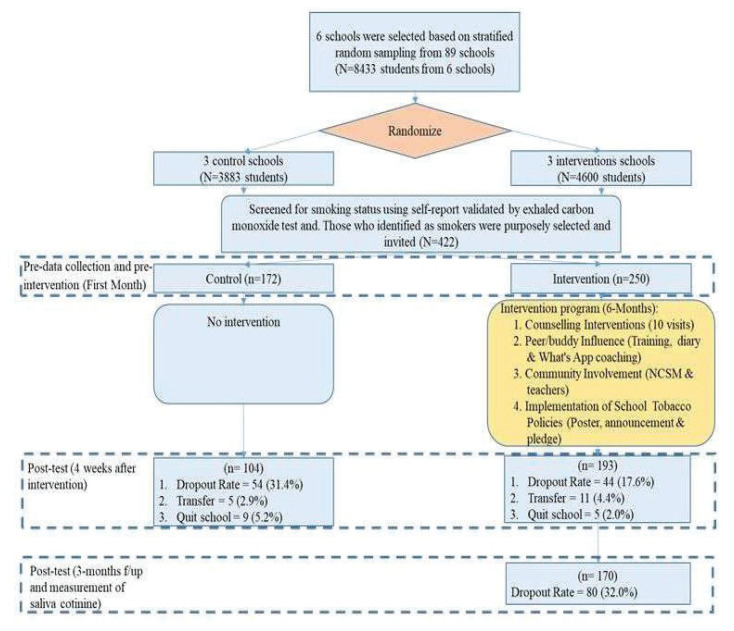
Flow chart for the FSSCP (CONSORT)

**Table 1:** Timeline and activities of the logic model application to the school-based FSSCP

Tasks	Duration
Pre-intervention preparations:	
Develop the proposal and meeting with NCSM	Aug 2017–Oct 2017
Preparation for MOE and UKM ethics approval and selection for schools for approval	Oct 2017–Nov 2017
Phase 1:	
Development of FSSCP’s training modules and survey measure; methods of data collection Develop the logic model for FSSCP	Oct 2017–Nov 2017
Training to intervention school counsellors and NCSM staff Perform the pilot tests for survey instruments	Dec 2017–Jan 2018
Conduct the CO screening and recruitment of smokers into the programme Pre-data collection and pre-intervention	Jan 2018–Feb 2018
Phase 2:	
Launch of the FSSCP	Feb 2018
Data analysis for pre-test	Feb 2018
Implementation of four interventions	Feb 2018–Aug 2018
Post-test and data analysis	Aug 2018–Sept 2018
Phase 3:	
Post-intervention evaluation (formative evaluation) of FSSCP In-depth interviews with counsellors, senior assistant student affairs and disciplinary teachers	Aug 2018–Sept 2018
Closing ceremony and graduation day	Oct 2018
Impact evaluation and report written for FSSCP	Nov 2018
Final report and presentation to FCTC unit, MOH for retention and sustainability of FSSCP	Dec 2018
Presentation to the task force committee, MOH	May 2019
Presentation to the Daily School Management Division, MOE	June 2019
Presentation to the Minister of Health, MOH	June 2019
Presentation to the Deputy Minister of Health, MOH, Chairperson for Electronic Cigarettes and Vaping Task Force	June 2019
Presentation to the Deputy Minister of Education, MOE	July 2019

Notes: CO = carbon monoxide; FCTC = Framework Convention on Tobacco Control; FSSCP = the Fit and Smart Adolescent Smoking Cessation Programme; MOE = Ministry of Education; MOH = Ministry of Health; NCSM = National Cancer Society of Malaysia; UKM = Universiti Kebangsaan Malaysia

**Table 2:** Demographic and socioeconomic characteristics of study participants

Characteristics	Pre-intervention (*n* = 422)			Post-intervention (*n* = 266)		

	Total (%)	Intervention (*n* = 250)	Control (*n* = 172)	Total (%)	Intervention (*n* = 168)	Control (*n* = 98)
Age (years old), 15.3 (SD = 1.24)						
13	34 (8.1)	19 (7.6)	15 (8.7)	25 (9.4)	12 (7.1)	13 (13.3)
14	93 (22.0)	71 (28.4)	22 (12.8)	60 (22.6)	49 (21.2)	11 (11.2)
15	90 (21.3)	73 (29.2)	17 (9.9)	58 (21.8)	48 (28.6)	10 (10.2)
16	123 (29.1)	86 (34.4)	37 (8.7)	81 (30.5)	59 (35.1)	22 (22.4)
17	82 (19.4)	1 (0.4)	81 (47.1)	42 (15.8)	0 (0.0)	42 (42.9)
Gender						
Male	381 (90.3)	215 (86.0)	166 (96.5)	237 (89.1)	142 (84.5)	95 (96.9)
Female	41 (9.7)	35 (14.0)	6 (3.5)	29 (10.9)	26 (15.5)	3 (3.1)
Ethnicity						
Bumiputera	342 (81.0)	239 (95.6)	103 (59.9)	220 (82.7)	164 (97.6)	56 (57.1)
Non-Bumiputera	80 (19.0)	11 (4.4)	69 (40.1)	46 (17.3)	4 (2.4)	42 (42.9)
Monthly household income (RM)						
≥ 1,000	27 (6.4)	22 (8.8)	5 (2.9)	20 (7.5)	16 (9.5)	4 (4.1)
1,001–4,000	347 (82.2)	210 (84.0)	137 (79.5)	216 (81.2)	141 (83.9)	75 (76.5)
≥ 4,000	48 (11.4)	18 (7.2)	30 (17.4)	30 (11.3)	11 (6.5)	19 (19.4)
Initiation age of smoking (years old)						
< 7	5 (1.2)	4 (1.6)	1 (0.6)	4 (1.5)	3 (1.8)	1 (1.0)
7–12	168 (39.8)	123 (49.2)	45 (26.2)	101 (38.0)	76 (45.2)	25 (25.5)
13–17	249 (59.0)	123 (49.2)	126 (73.2)	161 (60.5)	89 (53.0)	72 (73.5)
Smoking status						
Sole CC	114 (27.0)	91 (36.4)	23 (13.4)	34 (12.1)	22 (12.0)	12 (12.2)
Sole EC	178 (42.2)	87 (34.8)	91 (52.9)	9 (3.2)	9 (4.9)	0 (0.0)
Dual user (CC and EC)	130 (30.8)	72 (28.8)	58 (33.7)	43 (15.2)	20 (10.9)	23 (23.5)

Notes: SD = standard deviation; CC = conventional cigarettes; EC = electronic cigarette

**Table 3:** Quit rate at 4-week and 3-month follow-up in the intervention and control groups

Characteristics	Total (*n* = 266) *n* (%)	Intervention (*n* = 168) *n* (%)	Control (*n* = 98)* n (%)
Quitter at 4-week follow-up	119 (44.7)	95 (56.5)	24 (24.5)
Quitter at 3-month follow-up	–	70 (41.8)	–

Note:

* 3-month follow-up was not conducted for the control group

## References

[1] World Health Organization (WHO) and Centers for Disease Control and Prevention (2012). Malaysia Global School-based Student Health Survey (GSHS) [Internet].

[2] Ministry of Health (MOH) Malaysia (2016). Tobacco and e-cigarette report, 2016.

[3] Sargent JD, Gabrielli J, Budney A, Soneji S, Wills T (2017). Adolescent smoking experimentation as a predictor of daily cigarette smoking. Drug Alcohol Depend.

[4] Ministry of Health (MOH) Malaysia (2015). Pakej Program IMfree.

[5] Ministry of Health (MOH) Malaysia (2005). Tak Nak Merokok.

[6] Hassan H, Yusof N, Hashim RA (2014). Faktor rintangan ke atas kempen tak nak merokok. Jurnal Komunikasi Malays J Commun.

[7] Hum WL, Hsien CC, Nantha YS (2016). A review of smoking research in Malaysia. Med J Malaysia.

[8] Guo JL, Liao JY, Chang LC, Wu HL, Huang CM (2014). The effectiveness of an integrated multicomponent program for adolescent smoking cessation in Taiwan. Addict Behav.

[9] Fanshawe TR, Halliwell W, Lindson N, Aveyard P, Livingstone-Banks J, Hartmann-Boyce J (2017). Tobacco cessation interventions for young people. Cochrane Database Syst Rev.

[10] Jaber R, Taleb ZB, Bahelah R, Madhivanan P, Maziak W (2016). Perception, intention and attempts to quit smoking among Jordanian adolescents from the Irbid longitudinal study. Int J Tuberc Lung D.

[11] Owotomo O, Maslowsky J, Loukas A (2018). Perceptions of the harm and addictiveness of conventional cigarette smoking among adolescent e-cigarette users. J Adolesc Health.

[12] Helitzer D, Willging C, Hathorn G, Benally J (2009). Using logic models in a community-based agricultural injury prevention project. Public Health Rep.

[13] Department of Health and Human Services (2013). The logic model: the foundation to implement, study, and refine patient-centered medical home models. AHRQ Publication No 13-0029-EF.

[14] Gorini G, Carreras G, Bosi S, Tamelli M, Monti C, Storani S (2014). Effectiveness of a school-based multi-component smoking prevention intervention: the LdP cluster randomized controlled trial. Prev Med.

[15] Health Canada (2012). The Quit4Life (Q4L) youth cessation program facilitator’s guide.

[16] Towns S, DiFranza JR, Jayasuriya G, Marshall G, Smita Shah (2017). Smoking cessation in adolescents: targeted approaches that work. Paediatr Respir Rev.

[17] Leatherdale ST (2006). School-based smoking cessation programs: do youth smokers want to participate in these programs?. Addict Behav.

[18] Leatherdale ST, McDonald PW (2007). Youth smokers’ beliefs about different cessation approaches: are we providing cessation interventions they never intend to use?. Cancer Cause Control.

[19] Gabble R, Babayan A, DiSante E (2015). Smoking cessation interventions for youth: a review of the literature.

[20] Afifi RA, Makhoul J, El Hajj T, Nakkash RT (2011). Developing a logic model for youth mental health: participatory research with a refugee community in Beirut. Health Policy Plan.

[21] Gagnon RJ, Franz NK, Garst BA (2015). Factors impacting program delivery: the importance of implementation research in extension. J Hum Sci Extension.

[22] Shackleton N, Jamal F, Viner RM, Dickson K, Patton G, Bonell C (2016). School-based interventions going beyond health education to promote adolescent health: systematic review of reviews. J Adolesc Health.

[23] Mueller T, Tevendale HD, Fuller TR, House LD, Romero LM, Brittain A (2017). Teen pregnancy prevention: implementation of a multicomponent, community-wide approach. J Adolesc Health.

[24] Mihalic S, Fagan A, Irwin K, Ballard D, Elliot D (2004). Blueprints for violence prevention (NCJ 204274).

[25] Shakman K, Rodriguez SM (2015). Logic models for program design, implementation, and evaluation: workshop toolkit (REL 2015-057).

[26] Berkel C, Mauricio AM, Schoenfelder E, Sandler IN (2011). Putting the pieces together: an integrated model of program implementation. Prev Sci.

[27] Durlak JA, DuPre EP (2008). Implementation matters: a review of research on the influence of implementation on program outcomes and the factors affecting implementation. Am J Commun Psychol.

[28] Bucher JA (2010). Using the logic model for planning and evaluation: examples for new users. Home Health Care Management and Practice.

[29] Centers for Disease Control and Prevention (2007). Best practices for comprehensive tobacco control program—2007.

[30] Mishna F, Muskat B, Cook C (2012). Anticipating challenges: school-based social work intervention research. Child School.

[31] Bartlett R, Wright T, Olarinde T, Holmes T, Beamon ER, Wallace DJ (2017). Schools as sites for recruiting participants and implementing research. J Commun Health Nurs.

[32] Lamb J, Puskar KR, Tusaie-Mumford KJ (2001). Adolescent research recruitment issues and strategies: application in a rural school setting. J Ped Nurs.

[33] Faul F, Erdfelder E, Lang AG, Buchner A (2007). G* Power 3: a flexible statistical power analysis program for the social, behavioral, and biomedical sciences. Behav Res Methods.

[34] Bruzzese JM, Gallagher R, McCann-Doyle S, Reiss PT, Wijetunga NA (2009). Effective methods to improve recruitment and retention in school-based substance use prevention studies. J School Health.

[35] Grape A, Rhee H, Wicks M, Tumiel-Berhalter L, Joa Sloand EJ (2018). Recruitment and retention strategies for an urban adolescent study: lessons learned from a multi-center study of community-based asthma self-management intervention for adolescents. J Adolesc.

[36] Hurwitz LB, Schmitt KL, Fip Olsen MKJ (2017). Facilitating development research: suggestions for recruiting and re-recruiting children and families. Front in Psychol.

[37] Adikaram AS (2018). Being sensitive to the culture: challenges in researching sensitive topics in an Asian culture. Qual Res J.

[38] Ambrose BK, Rostron BL, Johnson SE, Portnoy DB, Apelberg BJ, Kaufman AR (2014). Perceptions of the relative harm of cigarettes and e-cigarettes among US youth. Am J Prev Med.

[39] Wheeler KC, Fletcher KE, Wellman RJ, Difranza JR (2004). Screening adolescents for nicotine dependence: the hooked on nicotine checklist. J Adolesc Healh.

[40] Alberta Health Services (n.d). Tobacco cessation toolkit: hooked on nicotine checklist (HONC) [Internet];.

[41] Price JH, Yingling F, Dake JA, Telljohann SK (2003). Adolescent smoking cessation services of school-based health centers. Health Educ Behav.

[42] Centers for Disease Control and Prevention (1994). Guidelines for school health programs to prevent tobacco use and addiction. MMWR.

[43] Harvey J, Chadi N, Canadian Paediatric Society, Adolescent Health Committee (2016). Strategies to promote smoking cessation among adolescents. Paed Child Health.

[44] Han Y, Kim H, Lee D (2016). Application of social control theory to examine parent, teacher, and close friend attachment and substance use initiation among Korean Youth. School Psychol Int.

[45] Schreuders M, Nuyts PA, van den Putte B, Kunst AE (2017). Understanding the impact of school tobacco policies on adolescent smoking behaviour: a realist review. Soc Sci Med.

